# Ethical Concerns About ChatGPT in Healthcare: A Useful Tool or the Tombstone of Original and Reflective Thinking?

**DOI:** 10.7759/cureus.54759

**Published:** 2024-02-23

**Authors:** Marina Z Kapsali, Efstratios Livanis, Christos Tsalikidis, Panagoula Oikonomou, Polychronis Voultsos, Aleka Tsaroucha

**Affiliations:** 1 Postgraduate Program on Bioethics, Laboratory of Bioethics, Democritus University of Thrace, Alexandroupolis, GRC; 2 Department of Accounting and Finance, University of Macedonia, Thessaloniki, GRC; 3 Department of General Surgery, Democritus University of Thrace, Alexandroupolis, GRC; 4 Laboratory of Experimental Surgery, Department of General Surgery, Democritus University of Thrace, Alexandroupolis, GRC; 5 Laboratory of Forensic Medicine & Toxicology (Medical Law and Ethics), School of Medicine, Faculty of Health Sciences, Aristotle University of Thessaloniki, Thessaloniki, GRC

**Keywords:** education, healthcare research, ethical framework, artificial intelligence, chatgpt

## Abstract

Artificial intelligence (AI), the uprising technology of computer science aiming to create digital systems with human behavior and intelligence, seems to have invaded almost every field of modern life. Launched in November 2022, ChatGPT (Chat Generative Pre-trained Transformer) is a textual AI application capable of creating human-like responses characterized by original language and high coherence. Although AI-based language models have demonstrated impressive capabilities in healthcare, ChatGPT has received controversial annotations from the scientific and academic communities. This chatbot already appears to have a massive impact as an educational tool for healthcare professionals and transformative potential for clinical practice and could lead to dramatic changes in scientific research. Nevertheless, rational concerns were raised regarding whether the pre-trained, AI-generated text would be a menace not only for original thinking and new scientific ideas but also for academic and research integrity, as it gets more and more difficult to distinguish its AI origin due to the coherence and fluency of the produced text. This short review aims to summarize the potential applications and the consequential implications of ChatGPT in the three critical pillars of medicine: education, research, and clinical practice. In addition, this paper discusses whether the current use of this chatbot is in compliance with the ethical principles for the safe use of AI in healthcare, as determined by the World Health Organization. Finally, this review highlights the need for an updated ethical framework and the increased vigilance of healthcare stakeholders to harvest the potential benefits and limit the imminent dangers of this new innovative technology.

## Introduction and background

Artificial intelligence (AI) is a leading technology in computer science that aspires to incorporate human behavior and intelligence into machines or systems by automating intellectual tasks normally performed by humans, using complex algorithms, such as machine learning and deep learning, to achieve this goal [1,2].

The current age, described as the era of the 4th Industrial Revolution [1], has been characterized by a huge technological event - the rise of AI. According to the vision of a collective of computer scientists in the Dartmouth Summer Research Program in 1956 [2], AI has emerged as a cutting-edge technology, and over the years, the basic theoretical concept of machines capable of thinking and behaving like humans has been put into action through the development of advanced computer programming and algorithmic systems. To date, AI has become a very popular subject not only in scientific literature [2], but it has also been progressively introduced in many areas of everyday life. Various types of AI, such as analytical, functional, interactive, textual, and visual AI, have been developed and put to trial, aiming to solve real-world problems. AI and its complex algorithms appear more and more frequently in a multitude of practical applications, including, among others, the sector of healthcare, offering new tools and possibilities in the diagnostic and therapeutic fields. Merely a few years ago, the coronavirus pandemic accelerated the expansion of AI applications in healthcare, as AI-related technologies have played a crucial role in this recent worldwide health crisis [3]. Introduced in November 2022, ChatGPT (Chat Generative Pretrained Transformer) is an AI chatbot, known for its user-friendly interface and has gained significant popularity among its users.

**Figure 1:**
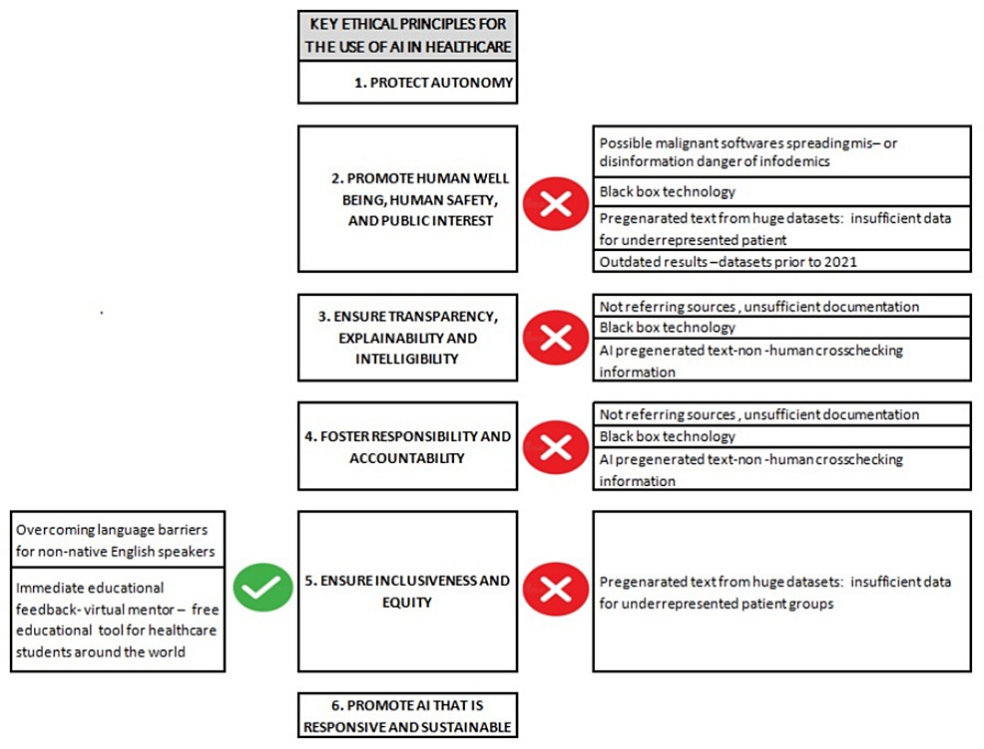
Schematic representation of ChatGPT features and their possible positive or negative effects on certain key ethical principles for the use of AI in healthcare. The potential positive and negative impacts of ChatGPT on crucial ethical principles [3] governing AI utilization in healthcare. This figure is an original work of the authors.

The increasing use of AI applications in clinical practice is changing the landscape of medicine, raising new ethical issues, and creating a pressing need for an updated bioethical framework. Lecadire et al. [3] documented a range of consequential risks associated with the implementation of AI in healthcare, including 1) patient injury due to AI errors, 2) improper use of medical AI tools, 3) bias in AI, 4) lack of transparency, 5) privacy and security issues, 6) accountability gaps, and 7) barriers to implementation [3]. In order to establish a comprehensive framework and underscore the principles of bioethics that should govern the use of AI in healthcare, official institutions, such as the European Parliament and the World Health Organization (WHO), have published relevant guidance. Specifically, in 2021, the WHO published the "Ethics and Governance of Artificial Intelligence for Health," formulating the six fundamental ethical principles that should govern the use of AI in healthcare (Table 1) [4]. As we embrace ChatGPT and its transformative force, we must also confront all the profound ethical concerns that accompany its integration in the ever-evolving landscape of healthcare and ensure that this AI application complies with these non-negotiable ethical mandates. By critically examining the present applications and the prospective uses of ChatGPT, this paper assesses the ethical dimensions of this emerging AI technology. Our analysis aims to juxtapose its key characteristics with the foundational principles established by the WHO for the ethical deployment of AI in the realm of healthcare (Figure 1).

**Table 1:** Key ethical principles for the use of artificial intelligence (AI) for healthcare [4].

Key ethical principles for the use of AI for health	
1	Protect autonomy
2	Promote human well-being, human safety, and public interest
3	Ensure transparency, explainability, and intelligibility
4	Foster responsibility and accountability
5	Ensure inclusiveness and equity
6	Promote AI that is responsive and sustainable

Methodology

We searched PubMed for relevant records using the following words or keywords: "ChatGPT," "healthcare," and "ethics." We used the search string (((Ethics) OR (Ethical Concerns) OR (Ethical Framework)) AND (Healthcare) AND (ChatGPT). The search conducted concluded on January 19, 2024, and yielded 93 records. It is to be noted that we encountered a limitation during the literature research. Since records containing AI-related ethical concerns might contain ChatGPT-related concerns, we conducted an additional search using the search string (((Ethics) OR (Ethical concerns) OR (Ethical framework)) AND (Healthcare) AND (Artificial Intelligence), which yielded 1,313 records. We conducted a screening of the 1,313 titles to identify additional records. We identified 17 records in addition to the 93 records.

Articles that contained abstracts, discussed ethical considerations pertaining to ChatGPT in healthcare, and were published in English were included in this review. Studies focusing on specific medical branches, specialties, areas, or topics were excluded from the study. Moreover, studies that referred to specific countries were excluded from the study. The data were extracted independently by all authors. In the first step, a title and abstract screening were performed. The initial screening was followed by a full-text screening. The authors evaluated all potentially eligible papers by reading the titles and abstracts. Following the full screening process, a total of 59 records were eligible to be included in the review. The articles deemed eligible for review were then further reviewed independently by two independent authors (MK and SL) using a thematic analysis. Any discrepancies were discussed and resolved in a consensus meeting.

The exclusion of records might have resulted in selection bias. While some studies focused on specific healthcare areas or countries, they might contain information that should not be excluded. It was not clear whether the inclusion or exclusion criteria were met. Any relevant uncertainty was discussed and resolved in a consensus meeting.

Quality assessment of the included papers

The authors made an effort to assess the methodological quality of the articles deemed eligible for review. As there are no specific quality assessment instruments for argument-based or normative ethics literature and the articles deemed eligible for review were indexed in the PubMed database, we fostered the assumption that the quality of these articles was reliable, to a greater or lesser extent. Nevertheless, to further confirm that assumption, we referred to "the '6Qs' of a method for appraising the quality of normative literature' offered by Mertz [5]. This tool is based on the "three possible strategies" and seems to be a method most promising in systematic reviews encompassing normative ethics literature. Furthermore, as the included articles employed not only normative ethics articles but also quantitative, qualitative, and mixed methods, the quality assessment of these articles was performed using the mixed methods appraisal tool (MMAT, version 2018), separately using the relevant sections of it [6]. Two independent authors (MK and SL) were assigned to assess the quality of the included articles. Initially, all articles were critically appraised for their quality by the first author (MK). Then, the last author (SL) checked the initial appraisal for reviewer inconsistency. A consensus meeting was held where both authors agreed upon the quality of the included articles.

## Review

ChatGPT: a new variable in the healthcare equation

In November 2022, OpenAI (OpenAI, L.L.C., San Francisco, CA, USA) launched an AI-based large language model (LLM) trained on massive text datasets in multiple languages with the ability to generate human-like responses to text input, called "ChatGPT" [7]. ChatGPT (Chat Generative Pretrained Transformer) is a chatbot based on neural networks capable of executing tasks by reading an enormous amount of existing human-generated text [8].

Given the high expectations and the promising prospects that followed its launch, ChatGPT was immediately put into action to ascertain its abilities, test its limits, and also point out its pitfalls. In the scientific community and academic world of healthcare, ChatGPT was received with controversial annotations and raised valid concerns regarding its potential in the fields of healthcare practice, education, and research [8]. Therefore, it almost comes as no surprise that, despite its very recent appearance in the scientific sphere, ChatGPT and its impact have already been the subject of many articles, reviews, studies, editorials, preprints, and other published manuscripts. At the time of the publication of this AI governance report from the WHO, ChatGPT was not part of the broader healthcare picture [4]. Nevertheless, this chatbot is currently trending in the AI universe, and although it is not an immediate or exclusive health-related application, it would be interesting to try to examine its compliance with these fundamental principles.

Ethical concerns in clinical practice

ChatGPT: Resulting in More Effective Clinicians?

The use of ChatGPT in clinical practice is still in a very primitive stage, and currently, ChatGPT is not intended by OpenAI for clinical use based on its terms of use [9]. Nonetheless, it has been the subject of studies discussing its potential contribution to healthcare and its transforming applications, especially in the diagnostic field, with innovative tools that can enhance the diagnostic and therapeutic process by directing differential diagnosis, safely predicting disease risk and outcomes [10], customizing healthcare plans that are adapted to patients' lifestyles and preferences [11,12], enhancing drug discovery, or even determining the imaging steps needed in breast cancer screening [13]. In addition, very recent studies suggest that this chatbot would also have the potential to reduce work hours for clinicians and assist in limiting cases of clinician burnout by contributing to medical bureaucracy writing, such as preauthorization letters to insurance companies, discharge summaries, or other documentation papers [11,14]. Moreover, ChatGPT can offer healthcare professionals the opportunity and the time to focus on the most creative part of medicine, as shown in the systematic review of Roman et al. [15], focusing on the applications of ChatGPT in neurosurgery issues and taking into account the potential risks [15].

Interestingly, studies using statistics have evaluated the capabilities and effectiveness of ChatGPT in the contexts of clinical practice, patients’ health literacy, and medical education. A cross-sectional survey showed that a majority (76.7%) of healthcare workers “believed ChatGPT could positively impact the future of healthcare systems” [16]. The authors concluded that most healthcare workers (75.1%) “were comfortable with incorporating ChatGPT into their healthcare practice” [16]. More precisely, it is stated that healthcare workers “perceived the chatbot to be useful in various aspects of healthcare, such as medical decision-making (39.5%), patient and family support (44.7%), medical literature appraisal (48.5%), and medical research assistance (65.9%)" [16]. However, the authors argued that healthcare workers’ concerns about “accuracy, reliability, and medicolegal implications of ChatGPT” serve as main barriers to successful implementation in healthcare settings [16]. Furthermore, Ayoub et al. conducted a cross-sectional study seeking to quantitatively analyze the ability of ChatGPT to “triage, synthesize differential diagnoses, and generate treatment plans” [17]. While the authors concluded that “ChatGPT has the potential to augment clinical decision-making," they highlighted that “more extensive research, however, is needed to ensure accuracy” [17]. Golan et al. conducted a study where they used statistics and concluded that while ChatGPT is a promising AI tool that has gained attention for its ability to evaluate the quality and readability of articles from websites, its current capabilities are less sufficient for doing so when compared to those of “human reviewers and readability assessment tools, such as Readable.com” [18].

ChatGPT: Resulting in Better Informed Patients?

Other studies also suggest that the chatbot’s conversational approach could contribute to patients’ education by expanding medical and scientific knowledge to the general public. This would facilitate a better understanding of complex information regarding diseases, therapeutic choices, and other healthcare issues, improving patient-doctor interaction [19-22]. As illustrated by Ayers et al. [23], not only could the clinicians save significant time in the electronic health record (EHR) by letting ChatGPT answer patient messages in the ambulatory HER [24], but the public was also more satisfied by the responses produced by the chatbot than those produced by real clinicians [23].

ChatGPT: A Tool That Comes With an Ethical Cost

While ChatGPT holds immense transformative potential in the field of medical practice, there remains uncertainty as to whether this transformation aligns with the intended direction. The indispensability of critical thinking skills for healthcare stakeholders raises doubts regarding the applicability of LLMs and an AI-based application, which may provide a mere catalog of predetermined answers and knowledge rather than encouraging professionals to evaluate diverse clinical dilemmas and tailor their approach to individual patients. LLMs have risks relating to the associations and biases of their training data and the propensity to generate “hallucinations,” meaning unfaithful or factually incorrect outputs [25]. Evidently, the adoption of clinical decisions and choices solely reliant on an LLM trained to simulate human-like responses through extensive data analysis poses significant risks to several key principles outlined by the WHO regarding the use of AI in healthcare. Limitations, such as reproducibility, fear of dehumanizing patient care [26], lack of transparency, risk of leaking patients' personal information and privacy data [27], and medicolegal implications according to the responses in a survey of healthcare workers in Saudi Arabia [28], such as accountability in cases of medical errors, further hinder the widespread utilization of ChatGPT as a clinical tool. Thus, the broader application of ChatGPT in healthcare practice remains distant.

Ethical concerns in healthcare education

ChatGPT: A New Age Mentor

Although there is still limited familiarization with its use, ChatGPT seems to have a really important impact on the future of healthcare education, and the expectations of healthcare trainees and professionals on ChatGPT-assisted training are already high, according to a survey in Taiwan [29]. Students in the medical or nursing fields could almost instantly have the answer to every single scientific question that would be created during their learning progress. Healthcare professionals could also easily classify, organize, and clarify the huge and constantly increasing amount of knowledge that they are supposed to memorize during their training years or their medical career [30]. Through its ability to generate human-like responses to student queries, ChatGPT could create a virtual tutor that adapts to the different learning styles of individual students, offering real-time feedback and keeping up with their personalized needs [7,31]. The ability of ChatGPT to provide accurate responses to knowledge-based medical questions of various types (multiple-choice, true/false, short-answer, short essay, and fill-in-the-blank types) has been the subject of recent quantitative analyses offering relevant statistical data [32]. Specifically, studies published as preprints evaluating the performance of ChatGPT have shown that ChatGPT had a successful result in three exams of the United States Medical Licensing Exam (USMLE) [33,34] and was able to perform at a human level on various datasets, including the USMLE (60.2%), MedMCQA (57.5%), and PubMedQA (78.2%). In addition, other reviews proved that the ChatGPT in a specialized multiple-choice exam performed at the level of an average first-year resident [7,35]. AI and language models could also be used to develop virtual training tools and stimulate cases and questions for various levels of training [36]. In addition to pure medical knowledge, this chatbot may even prove a useful tool in ethics education for healthcare professionals, helping them clarify all these complex terms and become familiar with the theory of bioethics [37].

ChatGPT can be useful in various aspects of medical education [38]. “ChatGPT and AI technology as a whole have the potential to revolutionize medical education” [39]. A cross-sectional web-based study showed that “medical students generally had a positive perception of using ChatGPT for guiding treatment and medical education" [40]. In a similar vein, a cross-sectional web-based study conducted by Alkhaaldi et al. showed that “despite limited experience and some ethics concerns, medical students were overall positive and optimistic about the future of AI in medical education" [39]. “However, some physicians are still unfamiliar with ChatGPT and are concerned about its benefits and risks" [40]. Another web-based cross-sectional survey conducted by Weidener and Fischer in German-speaking European countries showed a discrepancy between students’ interactions with ChatGPT and the representation of AI in their medical curricula (formal education). Most importantly, the authors highlight the urgent necessity of integrating not only the teaching of AI but also the teaching of AI ethics into the undergraduate medical curricula [41].

Searching for the scientific truth in ChatGPT: an imminent danger

ChatGPT in the area of healthcare education appears to already have a massive impact as an educational tool, and it is of great importance to ensure that medical education remains responsive to the needs of the digital era [42]. Nevertheless, rational concerns are raised regarding the quality of the knowledge offered and the compliance with the key principles of scientific writing ethics [43]. Pretrained-generated text and algorithm-based answers in scientific questions could lead to a) biased content, b) misleading or inaccurate information, c) the exclusion of results that are not commonly cited or results limited in time prior to 2021, and d) other misleading textual results [7,31,44]. Furthermore, it seems that ChatGPT's current capabilities are not sufficient for reliability in assessing the quality and readability of textual content [18,45], and it could easily provide incorrect, misleading health information [46]. In addition, the fact that all the information provided comes without reference to the sources is not only far from any accepted educational model but also dangerous, since it compromises the principles of human safety and public interest, transparency, explainability, and intelligibility and makes it almost impossible to attribute responsibility and accountability [47].

Ethical concerns for research

ChatGPT: An Additive in the Current Tools of Scientific Research?

In addition to healthcare education, the field of scientific research and academic writing is where this LLΜ comes in like a true wrecking ball. This textual AI technology is not only able to compose texts that are often characterized by a high level of original language, sufficient coherence between meanings, and the furthering of existing scientific understanding, but it can also assist in data collection when a particular query is posed [48] and in proposing optimal statistical methods for data analysis and transcribing the codes for use in R or Python [8]. On the one hand, for some researchers, this may seem exciting and revolutionary [49], since it could save time for the research parts that consume more effort from human intelligence (e.g., the focus on experimental design), as it derived from the systematic review, of ChatGPT in healthcare education, research, and practice using PRISMA guidelines conducted by Salam [7]. Moreover, ChatGPT could assist in creating queries for systematic reviews or other scientific content [50]. It could also be helpful in academic writing by sorting and managing the references and citations [51]. In addition, it seems that this chatbot would be of great use in solving practical issues of scientific writing by offering improvement in language and a better ability to express research ideas and results, especially for researchers who are non-native English speakers, simplifying and speeding up the publication process. This would be a decisive step toward promoting equity and diversity in research [52].

ChatGPT: A Tool Deprived of Fundamental Research Values

However, within certain academic circles, there is a prevalent conviction that such artificial intelligence technology will yield detrimental implications for medical research and the scientific literature [52,53]. There are concerns that ChatGPT may present inaccurate references, lack sufficient documentation, propagate incorrect information, or even potentially disseminate malicious disinformation disguised as scientific knowledge [8,54,55]. Several records have also mentioned that ChatGPT was referencing non-existing sources [56], functioning as a black-box technology, presenting a total lack of transparency. Furthermore, the acknowledgment of this LLM as an author would raise ethical obstacles and copyright issues since the pre-generated manuscripts that it creates include no citation of previous original sources and no attribution of credits to human authors or researchers. After ChatGPT was cited as an author of scientific papers for the first time [31,52] and taking into account all these concerns, prestigious journals, such as *Nature* and *Science*, rushed to adapt and modify their existing editorial policies by expressing their disapproval of accepting ChatGPT as an author of scientific papers [8,57]. In a worst-case scenario, without the ethical and legal framework, fluent and convincing AI-generated text composed by ChatGPT could easily be presented as human research work by certain researchers without the relevant disclosure and without having cited ChatGPT as an author [58]. That would be even more dangerous because the quality of the offered scientific information would not be able to be evaluated by the academic and scientific community as AI-generated, therefore unworthy and not credible, for all the reasons mentioned above. In that case, not only would plagiarism and originality issues arise, but it would also be a menace to the quality of the medical knowledge produced. In addition to the risk of plagiarism and inaccuracies, there is a chance of a potential imbalance in its accessibility between high- and low-income countries, if the software becomes available after payment [59].

Recommendations

Stahl and Eke emphasized the “lack of balance in considering ethical benefits and concerns about the use of ChatGPT (at present) [60]. Considering the balance between possible benefits and possible risks in light of a holistic ethics perspective seems to be necessary for developing effective guidelines [60,61]. De Angelis et al. stated, “There is a need for policy action to ensure that the benefits of LLMs are not outweighed by the risks they pose" [61]. Stahl and Eke regarded a good number of concepts “as having a high likelihood of being realized and a high level of social or ethical benefit" [60]. A long-lasting response to the question, “What measurable factors should be used to determine the threshold for deciding” whether possible risks outweigh possible benefits, should be regarded as a matter of high priority when discussing the ethical concerns raised by the use of ChatGPT in healthcare [62].

The recent acceleration in the development of AI and the widespread use of LLM applications, such as ChatGPT, demand drastic changes in healthcare education and necessitate the adaptation of medical training in order to adapt to this new AI era. In order to integrate these new technologies into clinical practice or make them a valuable research tool, we first have to ensure that healthcare professionals are familiar with their principles and aware not only of their potential but also of their dangers. Through short-term and long-term alterations in the educational medical system [42], future healthcare stakeholders should be able to navigate through this new and complex AI pathway. However, this navigation would be totally futile without an ethical compass. Therefore, now more than ever, we also have to make sure that medical education includes extensive familiarization with bioethics fundamental principles [43] and the ethical framework structured by competent institutions in order to ensure the safe, responsible, and beneficial use of AI potential.

Working toward this direction, the Panel for the Future of Science and Technology of the EU Parliament, driven by the accelerated and unsupervised expansion of AI in the medical field, launched mitigation measures addressing every potential main risk that has been identified by the use of AI in future healthcare [3]. With constant vigilance and proper adaptation into the corresponding ethical and legal framework, these measures, such as the establishment of regulatory agencies dedicated to medical AI, increased awareness of data privacy, and the demand for traceability and explainability as prerequisites for the certification of AI systems [3], could also be applicable for a safer use of ChatGPT.

While the use of AI in healthcare creates the need for stronger, transparent, and unbiased policy initiatives and regulatory interventions, the use of ChatGPT has contributed to the push for that need. Stronger policy initiatives and regulatory interventions need to be implemented on the international level to address issues that have global reach [60]. "Companies need to work with governments, regulators, and policymakers to address concerns around the use of ChatGPT in healthcare" [60]. Stahl and Eke emphasized “the need to consider the whole socio-technical AI ecosystem, not just specific issues” [60]. In the modern research landscape, researchers should familiarize themselves with ChatGPT [8]. In the healthcare research context, guidelines should be developed to secure “accountability, integrity, transparency, and honesty” [7,52]. Furthermore, in the modern context of healthcare education, more studies might evaluate and shed light on the potential impact of ChatGPT [7].

## Conclusions

The growing presence of AI, particularly ChatGPT, in healthcare is an undeniable reality that cannot be disregarded, and despite the emerging skepticism, instead of advocating for a blanket ban, it is essential to acknowledge its potential for transformation in teaching and learning while addressing associated risks through updated ethical frameworks. While ChatGPT can mimic human-like responses, it lacks critical and original thinking, which is the cornerstone of scientific progress. Our collective endeavors should be directed toward embracing the opportunities while effectively addressing the associated risks posed by this innovative technology by creating an updated ethical framework that effectively navigates this double-edged sword. Consequently, the trajectory of technologies like ChatGPT and whether they will be used as stepping stones or will turn out to be tombstones of healthcare education and research, hinges on our collective approach and ethical considerations.
